# Stacking Model‐Based Classifiers for Dealing With Multiple Sets of Noisy Labels

**DOI:** 10.1002/bimj.70042

**Published:** 2025-03-12

**Authors:** Giulia Montani, Andrea Cappozzo

**Affiliations:** ^1^ Data Reply srl Turin Italy; ^2^ Department of Statistical Sciences Università Cattolica del Sacro Cuore Milan Italy

**Keywords:** ensemble models, label noise, model‐based classification, multiple labels, supervised learning

## Abstract

Supervised learning in presence of multiple sets of noisy labels is a challenging task that is receiving increasing interest in the ever‐evolving landscape of healthcare analytics. Such an issue arises when multiple annotators are tasked to manually label the same training samples, potentially giving rise to discrepancies in class assignments among the supplied labels with respect to the ground truth. Commonly, the labeling process is entrusted to a small group of domain experts, and different level of experience and subjectivity may result in noisy training labels. To solve the classification task leveraging on the availability of multiple data annotators, we introduce a novel ensemble methodology constructed combining model‐based classifiers separately trained on single sets of noisy labels. Eigenvalue Decomposition Discriminant Analysis is employed for the definition of the base learners, and six distinct averaging strategies are proposed to combine them. Two solutions necessitate a priori information, such as the partial knowledge of the ground truth labels or the annotators' level of expertise. Differently, the remaining four approaches are entirely data‐driven. A simulation study and an application on real data showcase the improved predictive performance of our proposal, while also demonstrating the ability of automatically inferring annotators' expertise level as a by‐product of the learning process.

## 1 Introduction

The healthcare sector has experienced a notable proliferation in data availability, concomitant with the advancement of sophisticated statistical methodologies. It follows that it is only natural that these models are being utilized ever more frequently in the medical field (Chen et al. 2021). Among the diverse areas of application (see, e.g., Sidey‐Gibbons and Sidey‐Gibbons 2019, for an up‐to‐date review on the topic), we hereafter focus on supervised learning tasks. Supervised learning, also known as classification, entails training a model with labeled data to predict outcomes for new, unlabeled units. In the standard framework, it is assumed that the labels in the training set accurately identify the correct class to which each sample belongs. However, ground truth labels are challenging to obtain in many domains due to resource limitations, complex scenarios, and ambiguity (Frénay and Verleysen 2014). Specifically, manual labeling is common in the healthcare field, and relevant examples include, but are not limited to, medical images like X‐rays, MRIs, and histopathology slides (Cholleti et al. 2009; Zhu et al. 2014; Zhang et al. 2020, 2023). In most cases, the labeling process thus involves medical professionals carefully examining the samples and assigning labels to describe the observed features, abnormalities, or conditions present in the data. While human experts invaluably contribute to the labeling process, they may also introduce inconsistencies due to diverse domain knowledge, training backgrounds, and interpretations of visual cues. Medical professionals may encounter cases where a patient's symptoms or test results do not fit neatly into a predefined diagnostic category, making it difficult to assign a definitive label. Furthermore, a patient's medical condition might require costly and time‐intensive tests for a conclusive diagnosis, potentially placing a burden on patients, and causing delays in treatment. Therefore, it is essential to strike a balance between accurate data labeling and negative impact on patients' well‐being and resources. This can ultimately result in variations in how the same units are labeled by different annotators. The problem of training a classifier with multiple, and likely not entirely reliable, labels is known in the literature as “multilabel classification” or “classification with multiple sets of noisy labels,” and it will be the object of the present manuscript.

The generic multiclass multilabel classification issue is addressed, with the simpler problem of binary classification—commonly encountered in the medical context—readily included as a special case. In more detail, multi‐annotator supervised learning problems occur when labels are provided by multiple sources, such as several domain experts or a larger group of nonexperts, rather than a single labeler (Algan and Ulusoy 2021; Uma et al. 2021; Zhang et al. 2023). Such situations have received increased attention in recent years, primarily due to the proliferation of crowdsourcing services (Snow et al. 2008; Sorokin and Forsyth 2008; Welinder and Perona 2010). Indeed, the diverse insights and expertise of various contributors can enhance the quality of the labeling process. However, challenges arise as some annotators may be more reliable than others, some may act maliciously, and there may be varying levels of prior knowledge about the task. Furthermore, the quality may vary depending on the specific data instance being labeled. Standard methods to address these issues include filtering out low‐performing labelers and/or aggregating results from multiple annotators. In what follows, we will consider the latter strategy.

The proposed solution relies on an ensemble approach: by leveraging the annotations of multiple doctors, individual biases are reduced, and overall predictive performance is enhanced. The ensemble model is constructed by combining base learners separately trained on the noisy labels. Model‐based discriminant analysis is employed as base learner, with each classifier capturing distinct viewpoint embodying the expertise and inclinations of the corresponding annotator. For the purpose of combining the resulting models, different averaging strategies pursued directly on the estimated parameters of the model‐based classifiers are proposed. These techniques are tailored to specific scenarios, encompassing cases where expertise levels are only partially known or entirely unknown. The resulting pipeline capitalizes on the adaptability of ensemble density‐based models to effectively address the challenges posed by noisy labels in a multilabel scenario.

The rest of the paper is structured as follows. Section 2 highlights connections with previous methods and formally presents the proposed approach, providing the mathematical formulation and inferential aspects. Section 3 is dedicated to defining the weights used in the stacking process. Several options are detailed and, in conjunction with the definition of the ensemble procedure, they constitute the primary novel contribution of the present manuscript. Sections 4 and 5, respectively, present a simulation study and an application on gastrointestinal lesions detection, for which different opinions of seven clinicians must be leveraged upon. Section 6 concludes the manuscript and provides insights for future research directions.

The code developed for implementing the proposed methodology and replicating the results in the paper is freely available and can be accessed at https://github.com/GiuliaMontani/density‐based‐ensemble‐model.

## 2 Methodology

### 2.1 Related Work

Traditional supervised learning methods are designed to handle data with only one label set, making them inadequate for directly dealing with multiple labels associated with each sample. In presence of multiple noisy labels, practitioners have proposed several strategies for inferring the ground truth to enable the application of standard classifiers. The most commonly employed approach is majority vote, which aims at generating a single label, supposedly true, out of the multiple set of initially available ones (Lam 2000). While this approach is simple and straightforward, it discards valuable information such as the degree of uncertainty underlying the labeling process. To overcome this limitation, various proposals have been introduced in the literature to advance on the “Majority Vote” heuristic, mainly by making use of probabilistic approaches in which the unknown true labels are treated as latent variables (Dawid and Skene 1979; Jin and Ghahramani 2003; Whitehill et al. 2009; Raykar et al. 2009, 2010; Yan et al. 2010; Rodrigues et al. 2013; Zhang et al. 2013; Yan et al. 2014). More recently, kernel‐based methods and deep neural networks have also been devised to train a classifier with multiple sets of noisy labels (Gil‐Gonzalez et al. 2018; Guan et al. 2018; Sheng and Zhang 2019; Tanno et al. 2019; Gil‐Gonzalez et al. 2021; Herde et al. 2023; Li et al. 2023). These techniques are grounded in multiple architectures that collaboratively estimate both instance ground truth labels and annotators' performances; achieving this by learning and inferring interdependencies among instances, annotators, and their annotations (Zhang et al. 2016). Despite the groundbreaking achievements of deep learning methodologies in dealing with the multi‐annotator supervised learning problem, they still require a substantial amount of annotated data to be effectively utilized (Algan and Ulusoy 2021). Such a requirement may seldom be met in healthcare analytics. Therefore a different, perhaps simpler, path is pursued in this manuscript. We aim to circumvent the challenge of ground truth identification which may be difficult or even impossible when dealing with a limited number of annotators and data samples (McCluskey et al. 2021).

In detail, we rely on ensemble modeling for their renowned ability to combine information from various sources (Oza and Tumer 2008; Seni and Elder 2010). In such a way, different levels of expertise among annotators are properly and easily included in the learning process. To this aim, we propose to combine results from model‐based discriminant analysis, a well‐known and widely employed probabilistic framework for supervised classification (McLachlan 1992; Fraley and Raftery 2002). The most influential finding motivating this rationale stems from the recent work by Ahfock and McLachlan (2021), in which the authors show that soft labels can be more informative for the estimation of a classification rule compared to the single set of ground truth labels. The idea of staking density‐based models is not new in the literature. For example, Glodek et al. (2013) proposed to use an ensemble of Gaussian mixtures for probability density estimation. In a similar fashion, Bayesian model averaging was employed to postprocess results of model‐based clustering, to obtain a final partition based on a combination of unsupervised learners (Russell et al. 2015; Wei and McNicholas 2015). More recently, Casa et al. (2021) introduced an ensemble density‐based clustering procedure via a nonparametric formulation to avoid reliance on the single best model paradigm.

Despite some degree of connections with the above‐mentioned works, to the best of our knowledge no prior research has employed an ensemble of model‐based supervised learning models to address multiclass multilabel classification problems. The mathematical formulations for the single label set model‐based discriminant analysis, as well as the proposed ensemble procedure, are described in the following sections.

### 2.2 Model‐Based Discriminant Analysis

**FIGURE 1:**
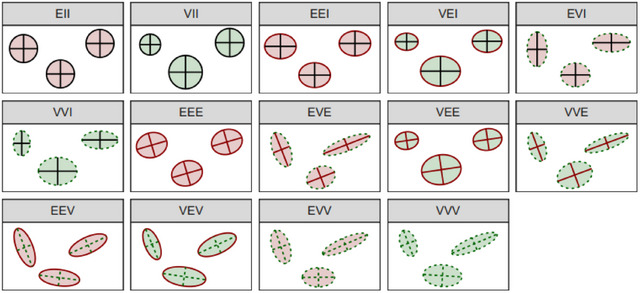
Ellipses of isodensity for each of the 14 Gaussian models obtained by eigendecomposition in case of three groups in two dimensions. Green (red) area denotes variable (equal) volume across components. Dashed green (solid red) perimeter denotes variable (equal) shape across components. Dashed green (solid red) axes denote variable (equal) orientation across components. Solid black perimeter denotes spherical shape. Solid black axes denote axis‐aligned orientation. Picture taken from Cappozzo et al. (2020a).

While reviewing the standard model‐based classification setting, let us already introduce the notation pertaining to the multilabel classification problem. Formally, consider a complete set of N learning observations (i.e., the training set, composed by G classes):

$$\begin{array}{ccc} \left(X,Y\right) & = & \{\left(x_{1},y_{11},\text{…},y_{1M}\right),\text{…},\left(x_{N},y_{1N},\text{…},y_{NM}\right);\;x_{n}\in R^{p},\\ & & y_{nm}\in \left\{1,\text{…},G\right\};\;\;n=1,\text{…},N,\;m=1,\text{…},M\}, \end{array}$$

where $x_{n}$ is a p‐dimensional continuous predictor and $y_{nm}$ identifies the gth label, g=1,…,G, assigned by the mth annotator to the nth data point. Notice that, contrarily to the standard framework, for every sample $x_{n}$ is associated a set of M, potentially different, class labels $y_{nm}$, m=1,…,M. Indeed, each of the M annotators classifies data point $x_{n}$ by assigning the class they deem correct. Let us denote with $x=\{x_{1},\text{…},x_{N}\}$ the features matrix while with $y_{m}=\left\{y_{1m},\text{…},y_{Nm}\right\}$ the set of noisy labels linked to the mth annotator. We employ a model‐based discriminant analysis classifier to separately model each pair $\left\{x,y_{m}\right\}$, m=1,…,M. In detail, we assume that the prior probability of group g for the mth annotator is $P\left(y_{m}=g\right)=\tau_{mg}$, with $\tau_{mg}>0$ and $\sum_{g=1}^{G}\tau_{mg}=1$. The gth class‐conditional density is modeled with a p‐dimensional Gaussian distribution with mean vector $\mu_{mg}\in R^{p}$ and positive semidefinite covariance matrix $\Sigma_{mg}\in PD\left(p\right)$ such that $x_{n}|y_{nm}=g∼N_{p}\left(\mu_{mg},\Sigma_{mg}\right)$. Therefore, the joint density of $\left\{x,y_{m}\right\}$ is given by

(1) $$\begin{array}{ccc} p(x,y_{m};\theta_{m}) & = & \prod_{n=1}^{N}\prod_{g=1}^{G}p\left(y_{m};\tau_{m}\right)p\left(x_{n}|y_{nm}=g;\mu_{mg},\Sigma_{mg}\right)\\ & = & \prod_{n=1}^{N}\prod_{g=1}^{G}{\left[\tau_{mg}\phi \left(x_{n};\mu_{mg},\Sigma_{mg}\right)\right]}^{1(y_{nm}=g)}, \end{array}$$

where $\phi (\cdot ;\mu_{mg},\Sigma_{mg})$ denotes the density of a multivariate normal random variable, $1(y_{nm}=g)$ is the indicator function meaning that observation n belongs to class g according to annotator m and $\theta_{m}=\left\{\tau_{m1},\text{…},\tau_{mG},\mu_{m1},\text{…},\mu_{mG},\Sigma_{m1},\text{…},\Sigma_{mG}\right\}$ is the collection of parameters to be estimated for the classifier associated to the mth annotator. The estimated density surfaces are ellipses centered at the mean $\mu_{mg}$, while their geometric properties are determined by the structure of $\Sigma_{mg}$. To achieve both flexibility and parsimony, Bensmail and Celeux (1996) introduced a family of models called Eigenvalue decomposition discriminant analysis (EDDA), an approach to design a classification rule based on a specific parameterization of $\Sigma_{mg}$. In detail, they assumed that the covariance matrix of the gth class can be factorized as follows:

(2) $$\Sigma_{mg}=\lambda_{mg}D_{mg}A_{mg}D_{mg}^{T},$$

where $D_{mg}$ is an orthogonal matrix of eigenvalues and corresponds to the orientation of the ellipse, $A_{mg}$ is a diagonal matrix and determines its shape while $\lambda_{mg}$ is a scaling parameter relative to the volume. Allowing the volume, shape, and orientation to be either equal (E) or different (V) across groups, with full (**E, **V), diagonal (**I), or spherical (*II) components, a family of 14 different models is obtained (see Figure 1 for a graphical representation and refer to Scrucca et al. (2016) for complete details). Particularly, it is worth noting that when it is assumed that all classes have the same covariance structure (EEE model) the well‐known linear discriminant analysis (LDA) approach is retrieved. On the other hand, the VVV specification forces different covariance structure within each group, thus defining a quadratic discriminant analysis (QDA) classification rule (Hastie and Tibshirani 1996; Qin 2018). Model in (1) is estimated through maximum likelihood, where depending on the chosen covariance structure either closed‐form solutions or iterative procedures are readily available (Bensmail and Celeux 1996). Software for fitting the EDDA routines is available within the mclust R package (Scrucca et al. 2016) and, if not specified otherwise, the best model among the 14 composing the family is automatically selected using the Bayesian information criterion (BIC, Schwarz 1978).

After separately estimating M EDDA models, one for each set of labels, our goal is to devise a meta‐learner by weighted averaging the contributions of each annotator. In doing so, we assume that each annotator provides their annotations independently. This assumption is commonly made and was originally postulated in the seminal work by Dawid and Skene (1979). The resulting ensemble procedure is described in the following subsection.

### 2.3 Ensemble of Model‐Based Classifiers

The main idea behind our staking paradigm is to leverage on the parametric nature of the EDDA family to construct a model‐based ensemble procedure whose parameters are a convex combination of those obtained by fitting separate models for each annotator. The M classifiers thus act as base learners, and we will investigate three different choices concerning their covariance structure: all‐equal in the G classes (LDA), all‐different (QDA), and the one automatically identified as best according to the BIC criterion (hereafter denoted with EDDA). After assigning a weight $w_{m}$ to each annotator m=1,…,M as described in Section 3, the construction of the ensemble model proceeds as follows:

- *Step 1 ‐ Fitting the base learners*:
  Given the training set $\left\{\left(x_{1},y_{11},\text{…},y_{1M}\right),\text{…},\left(x_{N},y_{1N},\text{…},y_{NM}\right)\right\}$ and the type of base learner (LDA, QDA, or EDDA), for each annotator a model‐based discriminant analysis is fitted, resulting in the ML estimates:
$${\hat{\theta }}_{m}=\left\{{\hat{\tau }}_{m1},\text{…},{\hat{\tau }}_{mG},{\hat{\mu }}_{m1},\text{…},{\hat{\mu }}_{mG},{\hat{\Sigma }}_{m1},\text{…},{\hat{\Sigma }}_{mG}\right\},\;m=1,\text{…},M.$$
- *Step 2 ‐ Weighted average of the base learners parameters:*
  The stacked model‐based classifier is obtained computing its parameters according to
(3) $$\begin{array}{cc} {\hat{\tau }}_{g} & =\sum_{m=1}^{M}w_{m}{\hat{\tau }}_{mg},\;g=1,\text{…},G, \end{array}$$
(4) $$\begin{array}{cc} {\hat{\mu }}_{g} & =\sum_{m=1}^{M}w_{m}{\hat{\mu }}_{mg},\;g=1,\text{…},G, \end{array}$$
(5) $$\begin{array}{cc} {\hat{\Sigma }}_{g} & =\sum_{m=1}^{M}w_{m}{\hat{\Sigma }}_{mg},\;g=1,\text{…},G, \end{array}$$
  where $w_{m}$ is the weight associated to the mth annotator, m=1,…,M.
- *Step 3 ‐ Update the sample labels according to the ensemble model:*
  Observations $x_{n}$, n=1,…,N, can then be reclassified to one of the G classes via the maximum a posteriori (MAP) rule:
(6) $$\max_{g\in 1,\cdots ,G}{\hat{z}}_{\mathrm{ng}}=\max_{g\in 1,\cdots ,G}\frac{{\hat{\tau }}_{g}\phi \left(x_{n};{\hat{\mu }}_{g},{\hat{\Sigma }}_{g}\right)}{\sum_{v=1}^{G}{\hat{\tau }}_{v}\phi \left(x_{n};{\hat{\mu }}_{v},{\hat{\Sigma }}_{v}\right)},$$
  where ${\hat{z}}_{ng}$ is the estimated posterior probability of $x_{n}$ belonging to class g according to the ensemble model.

The final output of the procedure includes the set of ensemble parameters ${\left\{{\hat{\tau }}_{g},{\hat{\mu }}_{g},{\hat{\Sigma }}_{g}\right\}}_{g=1}^{G}$ and the estimated posterior probabilities ${\hat{z}}_{ng}$. The stacked model‐based classifier can then be readily employed for predicting the class of an unlabeled observation via the MAP rule in Equation (6).

As an anonymous reviewer rightly pointed out, a remark is warranted regarding the use of the EDDA family in effectively tackling the considered multilabel classification problem. The strength of ensemble methods lies in combining predictions from different models, thereby reducing the overall variance (Breiman 1996a, 1996b). This is generally achieved by using “weak learners” in the averaging process to avoid overfitting. The classifiers in the EDDA family range from the flexible QDA (VVV model) to the simple spherical model with equal volume classes (EII model), accommodating a wide array of possibilities. Empirical evaluations (see Section 4) demonstrate that a model selected via the BIC criterion may choose diverse covariance structures across annotators. However, using strong classifiers as base learners in this context is not problematic because the risk of overfitting is mitigated by both the nature of the learning problem and the proposed techniques for determining weights (see Section 3). Specifically, on the one hand, in multilabel classification it is crucial to extract as much information as possible from each annotator. On the other hand, the distinction between reliable and less reliable annotators is automatically addressed in the second step of the proposed procedure. Here, less reliable annotators contribute less to the ensemble parameters calculation, justifying the usage of model‐based discriminant analysis as base learners.

We have outlined the steps necessary for building the ensemble model under the assumption of known weights. In practical situations, the weights need to be selected. Various strategies for this purpose are proposed in the following section.

## 3 On the Determination of the Annotators' Weights

In the previous section, we have formally defined a model‐based ensemble framework to handle classification with multiple sets of noisy labels. For making use of it, we must assign a weight $w_{m}$ to each annotator, that will ultimately influence their contribution to the estimation of the ensemble parameters, see Equations (3)–(5). The weight‐generating procedures at our disposal highly depend upon:

- the partial availability of ground truth labels,
- the expertise level of the annotators and the knowledge we have about it.

In the medical field, the former situation frequently arises when a group of patients undergoes specialized and informative diagnostic exams in addition to manual labeling by the annotators. Nonetheless, these exams may be invasive or time‐consuming so much so that only a subset of the training set will be subjected to them, leading to an incomplete view of the ground truth labels. A straightforward proposal for assigning weights to annotators under this scenario is described in Section 3.1.

Second, even when access to ground truth labels is not available, we may have some knowledge regarding the expertise level of the annotators. Considering expertise as a key factor ensures that higher weights are assigned to highly skilled annotators. However, in the specific context where annotators are doctors and labels correspond to disease diagnoses, objectively defining expertise level is not an easy task. It requires a comprehensive evaluation process that takes into account factors such as background, experience, and familiarity with the pathology. There must be guidelines to follow for the evaluation method to be fair and equitable. Otherwise, assigning weights based on subjective judgment can introduce bias. For instance, this may involve trusting highly skilled and trained doctors who lack competence in a specific condition under consideration. Similarly, it is important to avoid underestimating inexperienced doctors whose skills are not yet well‐known. A procedure for defining weights, assuming knowledge of the annotators' expertise levels, is described in Section 3.2.

Lastly, more often than not no prior information is available and the definition of the weights must thus be entirely data‐driven. In Section 3.3, we propose three options to do so: one is a somewhat naive equal weighting solution, whereas the other two provide pipelines to assign weights directly leveraging on the multiple set of noisy labels. In particular, the data‐driven computation of $w_{m}$, m=1,…,M, serves as a proxy for annotators' expertise, thus automatically inferring it as a by‐product of the learning process.

### 3.1 Partial Knowledge of Ground Truth Labels

In this context, the weights generation procedure is approached using a scoring system that assigns a score to each annotator. Formally, we are assuming that for a portion of the training set the ground truth labels are known, and we denote it with $y_{T}$. Specifically, let us define $N=N_{1}+N_{2}$, with $N_{1}$ and $N_{2}$ be the sample sizes for which the true labels are unknown and known, respectively. The label set can then be partitioned as $Y=Y_{1}\cup Y_{2}$, with

$$\begin{array}{ccc} & & Y_{1}=\left\{y_{nm};\;\;n=1,\text{…},N_{1},\;m=1,\text{…},M\right\},\\ & & Y_{2}=\left\{y_{nm};\;\;n=1,\text{…},N_{2},\;m=1,\text{…},\left(M+1\right)\right\}, \end{array}$$

where for the latter subset an extra set of ground truth labels $y_{T}$ is available. The proposed scoring system is reported in Algorithm 1, where we focus solely on the subset $Y_{2}$, comprising $N_{2}$ samples and M+1 labels for each statistical unit. The goal is to assess how often the labels assigned by the mth annotator align with the ground truth. The algorithm counts the number of agreements between the annotator's labeling and the true class, resulting in M integers that represent the associated scores $s=\{s_{1},\text{…},s_{M}\}$. A high score indicates that an annotator has consistently assigned the correct label, thereby reflecting their expertise in the labeling process. The so‐computed scores are then directly used to determine the weights in *Step 2* of the ensemble procedure (see Section 2.3), simply rescaling the resulting values:

(7) $${\hat{w}}_{m}=\frac{s_{m}}{\sum_{m=1}^{M}s_{m}},\;m=1,\text{…},M.$$

This objective and transparent scoring method leaves no room for subjectivity, as the evaluation relies solely on the agreement between the M manual labels and the true classes, assigning higher weights to annotators with higher performances in the scoring system. In so doing, we can confidently assess the expertise of each annotator and leverage this information in the ensemble model. At any rate, it goes without saying that this approach is only applicable when ground truth labels are available for at least a small proportion of the training set.

ALGORITHM 1 **Scoring system for partial knowledge of ground truth labels.**

**Table:** 

1:	Input: $N_{2}$, $Y_{2}$
2:	Initialize $s\in R^{M}=0$
3:	**for** $n\in N_{2}$ **do**
4:	**for** m∈M **do**
5:	**if** $y_{nm}==y_{nT}$ **then**
6:	$s_{m}+=1$
7:	**end if**
8:	**end for**
9:	**end for**
10:	Output: $s_{1},\text{…},s_{M}$

### 3.2 Knowledge of Expertise Level

The choice of weights outlined in this section is based on the a priori knowledge of the annotators' level of expertise. While such direct information may not always be readily available, previous annotator features or metadata could be accessible (Zhang et al. 2023). The proposed approach leverages on this to assign unequal weights that reflect the level of expertise. For the sake of simplicity, the method is presented by distinguishing only between annotators that are deemed experts and novices. Categorizing doctors into experts and novices is a valuable choice in the medical context as it allows to consider experience and preparation as discriminating factors. However, this binary categorization may lack granularity and not fully capture the spectrum of expertise levels among doctors that real‐world scenarios often involve. It is worth noting that the solution remains the same when multiple levels are taken into account. Without loss of generality and assuming a two‐level degree of expertise, the weights are computed as follows:

(8) $${\hat{w}}_{m}=\left\{\begin{array}{cc} \alpha & \text{if the}\;m\text{th annotator is an expert}\\ \beta & \text{if the}\;m\text{th annotator is a novice}, \end{array}\right.$$

under the constraint that $\sum_{m=1}^{M}w_{m}=1$. In the simplest case of M=2, this boils down to α+β=1. Typically β≤α, indicating that experts are generally assigned higher weights than novices. For instance, considering again the case with M=2, if one is very confident that the expert's opinions are significantly more valuable than the novice's, it is possible to set α=0.9 and β=0.1. When such a difference is not so obvious it is wiser to set values that are closer like α=0.7 and β=0.3.

This approach allows for an informed decision‐making process where the opinions of annotators with different levels of expertise are appropriately taken into account. Nonetheless, since expertise level is stated rather than estimated, it can be subjective and prone to biases, making it challenging to capture all nuances accurately. Therefore, if the distinction between the annotators is unknown or known but not completely trustworthy, it becomes crucial to establish a standardized and transparent evaluation system to minimize biases and ensure accurate categorization of doctors. On this wise, some data‐driven approaches are proposed in the following subsections.

### 3.3 Data‐Driven Approaches

In this section, three different strategies are outlined. The first one proposes a simple and intuitive solution, which is to assign the same weight to each annotator. Second, in order to capture potential differences between annotators, a scoring system based on majority voting (MV) is defined in Section 3.3.2. This system leverages the majority consensus among annotators to determine their individual weights, providing a more nuanced representation of their contributions. Lastly, in Section 3.3.3, an iterative algorithm is presented as an alternative to the previous scoring system. This iterative approach dynamically updates the annotators' weights refining the model parameters over subsequent iterations. Each approach is described in detail in the next subsections.

#### 3.3.1 Equal Weights

The most straightforward approach when no a priori information is available is to assign the same weight to each annotator:

(9) $${\hat{w}}_{1}=\cdots ={\hat{w}}_{m}=\cdots ={\hat{w}}_{M}.$$

An equal weights strategy provide a balanced approach that considers the collective opinion of all annotators, ensuring that no single one dominates the merged prediction, leading to a democratic decision‐making process. It assumes that annotators are equally trustworthy and have an equal level of expertise. Equal weights can lessen the effects of extreme or incorrect predictions even when the above‐mentioned condition is broken. However, if there is evidence suggesting that some annotators are more reliable or knowledgeable than others, alternative weighting schemes should be considered to reflect these differences.

#### 3.3.2 Majority Voting

The following approach to weights determination employs a data‐driven strategy based on the principle of Majority Vote (MV). MV theory is a decision‐making approach that revolves around selecting the class with the highest number of votes. This method is commonly used in ensemble learning algorithms, where multiple classifiers are combined to make a final decision (Breiman 1996a, 1996b). It is a simple yet effective technique that has been widely applied in various fields (Lam 2000). In healthcare, for instance, MV has been successfully utilized to improve diagnostic accuracy in, among others, lung cancer detection (Sünnetci and Alkan 2022) and Alzheimer diagnosis (Houria et al. 2023). These studies highlight that combining the results of individual classifiers in the simplest way can boost predictive performance. In our approach, we draw parallels to the concepts discussed in the standard MV framework while focusing on a distinct objective. Instead of reducing the multiple sets of labels into a single one through MV, we leverage MV in an earlier stage, that is during the determination of the weights to be assigned to each annotator. We make again use of a scoring system that will ultimately lead to the identification of the more reliable annotators. In detail, for each training unit we first identify the supposedly true label (hereafter denoted majority label) by applying MV to the labels provided by the M annotators. Having determined the set of majority labels, at each annotator is assigned a score $s_{m}$ reflecting the frequency with which their label $y_{nm}$ is equal to the majority label, for n=1,…,N. Similarly to what was done in Section 3.1, the so obtained scores are then rescaled to derive the final weights (see Equation 7).

Despite its conceptual simplicity, some peculiar cases need to be taken into account when devising the scoring system. To shed light on these potential issues, consider the synthetic example provided in Table 1, in which we report $y_{nm}\in \left\{1,2,3\right\}$ assuming to have N=3 units and M=5 annotators. The last column reports the majority labels.

**TABLE 1:** Synthetic example used to illustrate the scoring system. Majority vote is used for evaluating the skills of M=5 annotators (columns) for N=3 units (rows). The last column provides the majority labels computed via MV, while the last two rows report the score and associated weight obtained for each annotator, respectively.

	$y_{1}$	$y_{2}$	$y_{3}$	$y_{4}$	$y_{5}$	**Majority label**
	1	1	1	3	1	1
	3	2	3	1	3	3
	1	1	2	3	3	?
**Score**	**2**	**1**	**2**	**0**	**2**	
**Weight**	**0.285**	**0.145**	**0.285**	**0.0**	**0.285**	

Specifically, in the first line class 1 is identified as the majority label, thanks to the agreement of annotators 1,2,3, and 5. They are awarded one point, while the score of annotator 4 remains at zero. Moving on to the second row, the majority label is class 3 and, following the same logic previously applied, the score of annotators 1,3, and 5 increase of one unit. In the third line, a deadlock occurs. Class 1 and 3 receive two votes each. No true label can be assigned and therefore no score is awarded. This situation highlights a potential drawback in the MV criterion, as conflicts among annotators are ignored in the score computation. Even if there is substantial disagreement among annotators, MV would still compute a majority label, without considering the uncertainty introduced by the conflicting annotations. In Table 1, we can observe this limitation in action: 80% of the annotators agree that the label associated with the first sample is 1, while only 60% agree that the label to be assigned to the second unit should be 3. In both situations, the majority vote rule identifies a majority label. To mitigate this drawback, one option would be to set a threshold for the minimum percentage of agreement required to actually compute the majority label. This threshold can vary depending on the application and the desired level of agreement. A value that is extremely close to 100% may make it difficult to achieve the required level of agreement, increasing the likelihood of obtaining a score of 0 for annotator m, effectively resulting in the parameters estimated by the mth base learner being ignored in the ensemble model. In a medical setting, no annotator is expected to be completely unreliable, so we do not want to neglect the contribution of any of them. At any rate, the majority vote strategy offers a simple and interpretable approach for generating weights consistent with annotators' expertise without any a priori knowledge. A last and more refined iterative procedure is presented in the next subsection.

#### 3.3.3 Iterative Algorithms

The final procedure we develop for assessing the annotators' contribution to the ensemble learner involves an iterative algorithm, where weights and sample labels are sequentially refined until no more changes occur. Such a strategy stems from previous proposals employed in the binary classification settings (Zhang and Obradovic 2010, 2011), which we extend to handle the multiclass framework. Our solution begins by computing the M base learners and an initial set of labels ${\hat{y}}^{0}=\left\{{\hat{y}}_{1}^{0},\text{…},{\hat{y}}_{N}^{0}\right\}$ using MV (see Section 3.3.2). Subsequently, the iterative phase alternates a weights generation step through the scoring system of Section 3.1, followed by the reestimation of the ensemble model, which is then used to compute a new set of labels ${\hat{y}}^{t}$, at iteration t, by means of the MAP rule of Equation (6). The complete procedure is outlined in Algorithm 2. Notice that the process of fitting the model‐based classifiers need not be repeated at each iteration, as the M base learners remain constant throughout the iterative process and only the weights and the estimated labels are updated.

ALGORITHM 2 **Scoring system via iterative algorithm.**

**Table:** 

1:	Input: $\left(X,Y\right)=\left\{\left(x_{1},y_{11},\text{…},y_{1M}\right),\text{…},\left(x_{N},y_{1N},\text{…},y_{NM}\right)\right\}$
2:	Estimate the M base learners as per Step 1 of Section 2.3
3:	Initialize ${\hat{y}}^{0}=\left\{{\hat{y}}_{1}^{0},\text{…},{\hat{y}}_{N}^{0}\right\}$ using MV rule
4:	**while** ${\hat{y}}^{t}\neq {\hat{y}}^{t-1}\;\left|\right|\;t\leq \text{maxIt}$ **do**
5:	$s^{t}=0$
6:	**for** n∈N **do**
7:	**for** m∈M **do**
8:	**if** $y_{nm}=={\hat{y}}_{n}^{t-1}$ **then**
9:	$s_{m}^{t}+=1$
10:	**end if**
11:	**end for**
12:	**end for**
13:	Compute ${\hat{w}}_{m}^{t}=s_{m}^{t}/\sum_{m=1}^{M}s_{m}^{t}$, m=1,…,M
14:	Update the ensemble learner as per Step 2 of Section 2.3
15:	Compute ${\hat{y}}^{t}$ as per Step 3 of Section 2.3
16:	**end while**
17:	Output: $s_{1}^{t},\text{…},s_{M}^{t}$

The primary advantage of the iterative scoring system is its wholly data‐driven nature and its capacity to evaluate annotators based on their scores. Nonetheless, in its current form it does come with a drawback, as the information about the uncertainty of the estimated label is not taken into account. Indeed, if the label provided by the mth annotator agrees with the estimated one for observation n, then one point is directly awarded to the annotator. To address this issue, a different scoring system is also investigated where instead of assigning a unit score for every agreement between ${\hat{y}}^{t}$ and $y_{m}$, we make directly use of the probability of class assignment estimated for the mth annotator. The utilization of probabilities instead of integer values enables the distinction between varying levels of confidence among annotators. To illustrate this approach, consider the simple example in Table 2 involving M=3 annotators, N=1 observation, and G=3 classes. The last column provides the estimated label ${\hat{y}}_{1}^{t-1}$ computed at iteration t−1, while the last two rows report the scores $s_{m}^{t}$ assigned following the two variations of the iterative algorithm, hereafter denoted ItAlg1 and ItAlg2, respectively.

**TABLE 2:** Synthetic example used to illustrate the difference between the scoring system proposed in iterative Algorithm 2 and its variation. The last column provides the estimated label. In the first row are reported the class probability membership for each annotator, in the second and third rows the scores obtained adopting the two different scoring procedures.

	$y_{1}$	$y_{2}$	$y_{3}$	**Estimated label**
${\hat{z}}_{n}$	(0.8,0.1,0.1)	(0,5,0.3,0.2)	(0.4,0.5,0.1)	1
$s_{m}^{t}$ according to ItAlg1	1	1	0	
$s_{m}^{t}$ according to ItAlg2	0.8	0.5	0	

In this simple example, annotators 1 and 2 receive the same score using the first method, but different scores using the second. We can distinguish between different confidence levels by allocating soft labels (i.e., probabilities), rather than hard labels as scores. Notice that, due to incorrect labeling, annotator 3 gets a score of 0 in both cases. While the choice between using soft and hard scores may not significantly impact the weight computation (see Sections 4 and 5), we find both approaches useful for achieving a finer‐grained breakdown of the different levels of expertise among the annotators.

The present section has been devoted to the construction and justification of several proposals for objectively determining the weights associated to the M annotators composing the ensemble model. The resulting taxonomy is summarized in Table 3, where the acronyms, names, and required level of a priori information are conveniently reported. All approaches will be compared and tested in synthetic and real‐data scenarios in the upcoming sections.

**TABLE 3:** Overview of the weights generation strategies proposed in Section 3 for constructing the ensemble model.

**Acronym**	**Strategy name**	**Strategy description**	**Required a priori information**
**PGT**	Partial Ground Truth	Scoring system with subset of ground truth labels	Subset of ground truth
**EN**	Experts VS Novices	Scoring system with knowledge of expertise level	Expertise level
**E**	Equal weights	Fixed weights equal to 1/M	None
**MV**	Majority Vote	Scoring system with subset of labels	Annotators agreement level
**ItAlg1**	Iterative Algorithm Opt. 1	Iterative scoring algorithm with integer scores	None
**ItAlg2**	Iterative Algorithm Opt. 2	Iterative scoring algorithm with probabilities scores	None

## 4 Simulation Studies

We conduct a simulation study to evaluate the effectiveness of the density‐based ensemble model proposed in Section 2. Our main goal is to evaluate the performance of the novel methodology using various weight determination strategies, alongside base learners trained on a single set of noisy labels. In addition, we aim to compare our approach with state‐of‐the‐art alternatives for addressing multilabel classification. The models are evaluated considering both their predictive accuracy and their estimation bias with respect to the true generated parameters.

### 4.1 Data‐Generating Process

#### 4.1.1 Feature Space

The simulation considers a data set composed by G=3 classes with N=150 bivariate samples. In the first scenario, the features $x_{n}$, n=1,…,N are generated from three equiprobable Gaussian distributions $\phi (\mu_{g},\Sigma_{g})$ with mean vectors

$$\mu_{1}=\left(2,2\right),\;\mu_{2}=\left(0,0\right),\;\mu_{3}=\left(-2,-2\right),$$

and covariance matrices

$$\Sigma_{g}=I_{3},\;g=1,\text{…},3.$$

**FIGURE 2:**
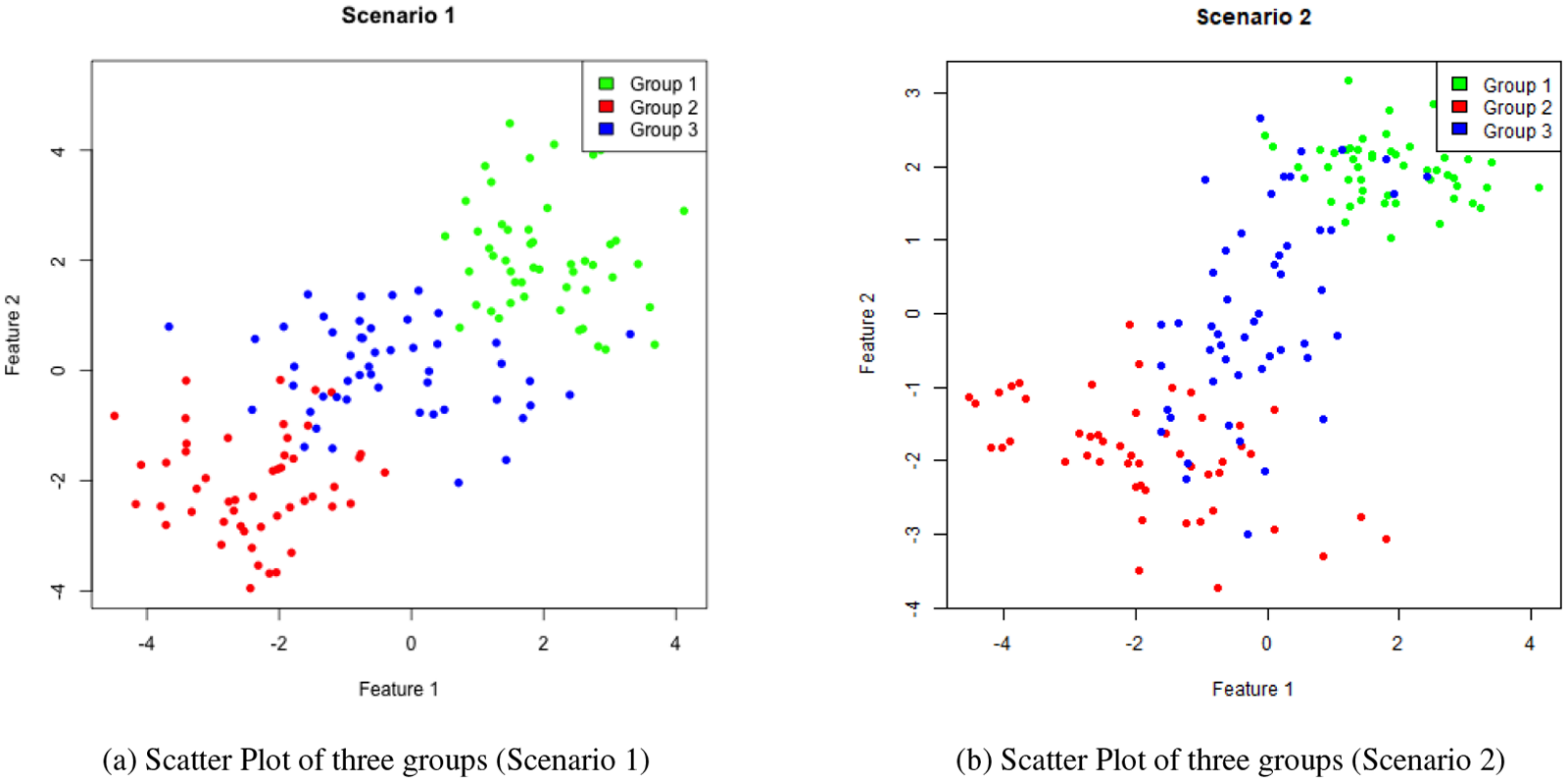
Synthetic data generated from Gaussian distributions: in Panel (a) the covariance structures are the identity matrices $I_{2}$, while in Panel (b) the covariance structures are class‐wise different. Colors represent the ground truth labels.

In a second scenario, we assume class‐wise different correlation among the features, with the covariance matrices generated using the clusterGeneration R package. In Figure 2, synthetic samples under the two considered data‐generating processes are graphically reported.

#### 4.1.2 Multiple Noisy Labels

We consider in the study a panel of M=7 annotators and we make use of the Multinomial distribution for generating their associated set of noisy labels. That is, for each training unit n and annotator m we have:

(10) $$Y_{nm}∼\text{Multinomial}\left(1,p\right),$$

where the parameter vector $p=\{p_{1},\text{…},p_{G}\}$, satisfying $\sum_{g=1}^{G}p_{g}=1$ and $p_{g}\geq 0$ for all g, reports the probabilities of the mth annotator to assign the nth observation to the gth class. Clearly, in the absence of uncertainty in the labeling process, the label corresponding to the observation of the true class being g would be sampled from a Multinomial distribution with a parameter vector containing all zeros except for a 1 in the gth position, that is, $p=e_{g}$, where $e_{g}$ identifies a vector of length G with entries all equal to 0 but a 1 in the gth position. Nonetheless, we are interested in simulating a scenario in which labels are subjected to noise. To capture the variability introduced by the manual annotation process, we adopt a Dirichlet Multinomial distribution (Johnson et al. 1997) to simulate the M sets of noisy labels. This model extends the Multinomial distribution by integrating a Dirichlet prior. The probability density function has the following form:

(11) $$\begin{array}{ccc} Dir(p|\alpha ) & = & \frac{1}{Beta(\alpha )}\prod_{g=1}^{G}p_{g}^{\alpha_{g}-1}\;\text{where}\\ Beta(\alpha ) & = & \frac{\prod_{g=1}^{G}\Gamma \left(\alpha_{g}\right)}{\Gamma (\sum_{g=1}^{G}\alpha_{g})}. \end{array}$$

**FIGURE 3:**
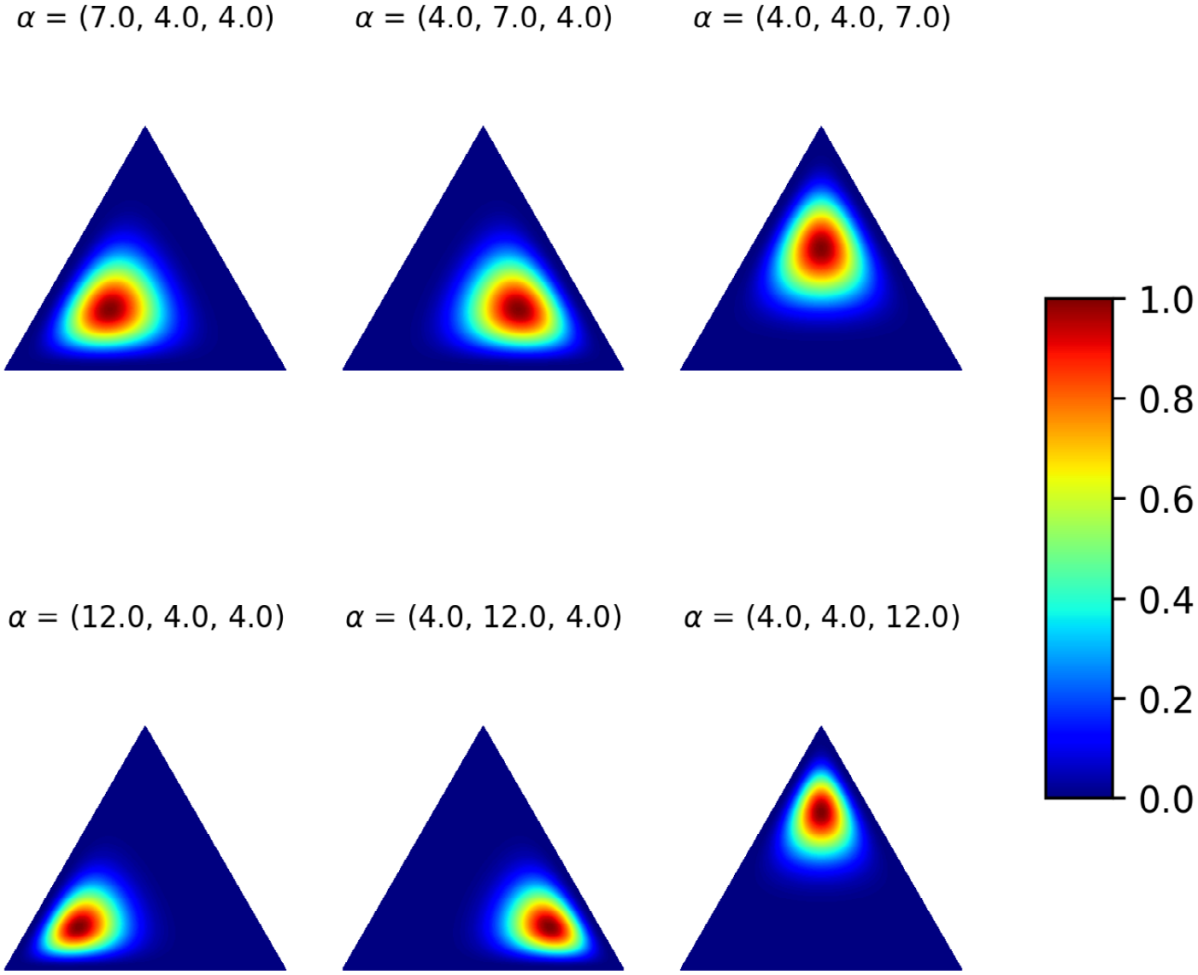
Dirichlet distribution on a two‐dimensional simplex for different value of the hyperparameter α, visualized with a graded color scale ranging from blue (low probability) to red (high probability). The triangle represents the possible combinations of G=3 classes. In the first row, the distribution describes novices behavior within the three groups while the second row denotes the experts annotation process.

In our simulation setting, we consider G=3 classes, resulting in a three‐dimensional Dirichlet distribution with parameters $\alpha =(\alpha_{1},\alpha_{2},\alpha_{3})$ that govern both the mean and variance of the distribution. The generating process for the noisy labels thus reads as follows:

(12) $$\begin{array}{ccc} & & Y_{nm}∼\text{Multinomial}\left(1,\text{Dir}\left(p|\alpha_{m}\right)\right),\\ & & m=1,\text{…},M,\;n=1,\text{…},N, \end{array}$$

where $\alpha_{m}=\left(\alpha_{1m},\alpha_{2m},\alpha_{3m}\right)$. Different combinations of $\alpha_{m}$ values lead to distributions that are skewed or asymmetric, thus reflecting the different degree of confidence in the labeling process by the M annotators. Specifically, larger values of $\alpha_{gm}$ result in a more concentrated distribution, meaning that higher probabilities are assigned to group g by the mth annotator. In the healthcare context, annotators all have a good base of experience and therefore when the true class is the gth one we correspondingly set $\alpha_{gm}$ to be the highest value for all m, indicating that the gth class is the most likely outcome. Nonetheless, by increasing or decreasing $\alpha_{gm}$ we can simulate different expertise levels among the M annotators. In our simulated scenario, we assume there are two distinct levels of expertise: four annotators are classified as experts, while the remaining three are designated as novices. This setup mirrors the real‐data analysis discussed in Section 5. In Figure 3, we report the Dirichlet distributions employed for simulating the novice (first row) and expert (second row) annotation processes.

#### 4.1.3 Additional Simulation Details

As previously reported, we consider three different covariance structures for the base learners of our ensemble procedure: all‐equal in the G classes (LDA), all‐different (QDA), and the one automatically identified as best according to the BIC criterion (EDDA). For each scenario, we run the simulation S=50 times. We make use of the acronyms introduced in Table 3 for referring to the different weights determination strategies. As some of them require a priori information, we hereafter describe the choices made in the simulation settings. The first ensemble model PGT (Partial Ground Truth) requires knowledge of a subset of ground truth label. In our simulation, we assume a 10% of ground truth labels known, this choice strikes a balance between having a modest amount of ground truth information to guide the scoring process and maintaining a degree of uncertainty to mimic real‐world scenarios. Similarly, for the subsequent EN (Experts VS Novices) model, we consider a setting where experts are assigned 80% of the total contribution to the weights. Indeed, during the simulation study we know which annotators are experts, but in practice one must rely on the judgment of a third party. When it comes to data‐driven approaches instead, apart from MV, they generally do not necessitate the configuration of specific hyperparameters. Indeed, the weights are either set to be all‐equal or generated through tailored scoring algorithms. MV, while also being a fully data‐driven method, requires the definition of a threshold value on the agreement level (see Section 3.3.2). We set the threshold at approximately 80% of the total number of annotators M. Namely, five out of seven annotators must agree to consider the majority label in the scoring system.

Lastly, we compare the predictive performance of our method with three latent variable models designed for learning classifiers from multiple annotators:

- *Dawid and Skene*: the pioneering work on maximum likelihood estimation of observer error rates considering only the annotators' frequencies of response, as proposed in Dawid and Skene (1979),
- *Raykar*: a probabilistic approach for supervised learning that jointly learns the classifier, the annotator accuracy, and the actual true label, as proposed in Raykar et al. (2010),
- *Rodrigues*: a probabilistic model that explicitly handles the annotators' reliabilities with latent variables without relying on the estimation of the unknown ground truth labels, as proposed in Rodrigues et al. (2013).

A Julia (Bezanson et al. 2017) implementation of the aforementioned approaches can be found in the fmpr/LogReg‐Crowds GitHub repository.

### 4.2 Simulation Results

In this section, we present the results of the simulation study. At each repetition of the simulated experiment, we randomly split the synthetic data set of 150 observations into a training set of 100 data points and a test set of 50 units. We conduct a stratified train‐test split to ensure that both subsets maintain the same proportion of different classes as the original dataset.

#### 4.2.1 Analysis of Models Performance

To comprehensively evaluate the models performance, we adopt a diverse range of metrics, encompassing accuracy and parameter estimation error. The overall results, in terms of both predictive power and the assessment of the estimated model parameters for the methodology proposed in Section 2, are presented in Table 4. For the competing methods introduced at the end of Section 4.1.3, we evaluate them solely based on their predictive ability. The results are presented in Table 5.

**TABLE 4:** Average accuracy, ${\left||\Delta \Sigma |\right|}_{F}$ and MSE for the S=50 repetitions of the simulated experiments, varying covariance structure, weights generation procedure, and ensemble strategy for the model proposed in Section 2. The first section collects the result of the simulation in Scenario 1 (see Figure 2a), while the second section reports results of Scenario 2 (see Figure 2b). Standard deviations for accuracies are reported in parentheses. Each row corresponds to a different model.

	Scenario 1							Scenario 2						
	ACC			${\left||\Delta \Sigma |\right|}_{F}$			MSE	ACC			${\left||\Delta \Sigma |\right|}_{F}$			MSE
	LDA	QDA	EDDA	LDA	QDA	EDDA		LDA	QDA	EDDA	LDA	QDA	EDDA	
**GT**	0.891 (0.05)	0.889(0.05)	0.891 (0.05)	0.11	0.36	0.11	0.21	0.859 (0.05)	0.880 (0.05)	0.871 (0.05)	1.21	0.39	0.54	0.21
**E1**	0.724 (0.13)	0.691 (0.13)	0.664 (0.11)	3.22	4.65	4.63	1.36	0.705 (0.12)	0.655 (0.11)	0.652 (0.12)	3.38	4.69	4.71	1.36
**E2**	0.733 (0.15)	0.705 (0.09)	0.673 (0.13)	3.15	4.55	4.52	1.30	0.718 (0.13)	0.691 (0.10)	0.671 (0.12)	3.32	4.58	4.60	1.30
**E3**	0.709 (0.11)	0.670 (0.11)	0.644 (0.11)	3.21	4.63	4.61	1.36	0.693 (0.11)	0.655 (0.10)	0.637 (0.10)	3.37	4.65	4.69	1.37
**E4**	0.745 (0.12)	0.694 (0.10)	0.676 (0.10)	3.16	4.56	4.53	1.31	0.725 (0.11)	0.683 (0.11)	0.672 (0.11)	3.33	4.59	4.62	1.32
**N1**	0.633 (0.14)	0.591 (0.12)	0.569 (0.13)	3.47	4.97	4.97	1.58	0.613 (0.15)	0.579 (0.11)	0.550 (0.12)	3.62	5.03	5.05	1.58
**N2**	0.678 (0.17)	0.602 (0.14)	0.581 (0.16)	3.46	5.00	4.95	1.56	0.656 (0.15)	0.583 (0.16)	0.571 (0.15)	3.60	5.05	5.02	1.56
**N3**	0.647 (0.13)	0.579 (0.12)	0.562 (0.11)	3.46	4.96	4.96	1.57	0.650 (0.12)	0.573 (0.11)	0.566 (0.12)	3.60	5.00	5.02	1.57
**PGT**	0.758 (0.08)	0.740 (0.07)	0.758 (0.07)	4.70	4.70	4.70	1.37	0.724 (0.07)	0.712 (0.09)	0.720 (0.06)	4.78	4.73	4.78	1.37
**EN**	0.765 (0.07)	0.744 (0.08)	0.765 (0.07)	4.65	4.65	4.65	1.34	0.741 (0.06)	0.715 (0.09)	0.740 (0.06)	4.73	4.67	4.73	1.34
**E**	0.749 (0.07)	0.744 (0.08)	0.749 (0.07)	4.74	4.74	4.74	1.39	0.727 (0.07)	0.716 (0.10)	0.726 (0.07)	4.81	4.76	4.81	1.39
**MV**	0.755 (0.07)	0.740 (0.08)	0.755 (0.07)	4.73	4.73	4.73	1.38	0.728 (0.07)	0.715 (0.09)	0.727 (0.07)	4.80	4.75	4.80	1.39
**ItAlg1**	0.750 (0.07)	0.742 (0.08)	0.750 (0.07)	4.71	4.71	4.71	1.37	0.725 (0.06)	0.715 (0.09)	0.725 (0.06)	4.78	4.74	4.78	1.38
**ItAlg2**	0.748 (0.07)	0.742 (0.08)	0.748 (0.07)	4.70	4.71	4.70	1.37	0.723 (0.07)	0.715 (0.09)	0.723 (0.07)	4.78	4.74	4.78	1.38

**TABLE 5:** Average accuracy for the S=50 repetitions of the simulated experiments for the competing models described in Dawid and Skene (1979), Raykar et al. (2010), and Rodrigues et al. (2013). The first section collects the result of the simulation in Scenario 1 (see Figure 2a), while the second section reports results of Scenario 2 (see Figure 2b). Standard deviations are reported in parentheses.

	Scenario 1			Scenario 2		
	**Dawid and Skene**	**Raykar**	**Rodrigues**	**Dawid and Skene**	**Raykar**	**Rodrigues**
**ACC**	0.689 (0.061)	0.695 (0.036)	0.703 (0.038)	0.689 (0.061)	0.695 (0.036)	0.703 (0.042)

We first look at the predictive power of the models by assessing the accuracy on the test set. Given that the simulation is repeated S=50 times, the values in Tables 4 and 5 correspond to the average accuracy, denoted with ACC. Alongside, we provide the standard deviation (SD), which quantifies the spread of accuracy across the simulations. Mathematically, the two quantities are defined as follows:

(13) $$\begin{array}{cc} \text{ACC} & =\frac{1}{S}\sum_{s=1}^{S}ACC_{s}, \end{array}$$

(14) $$\begin{array}{cc} \text{SD} & =\sqrt{\frac{1}{S-1}\sum_{s=1}^{S}{\left(ACC_{s}-ACC\right)}^{2}}, \end{array}$$

where $ACC_{s}$ is the accuracy of the sth simulation run. The first three columns of Table 4 report the numerical values for ACC and SD for the first scenario (see Figure 2a). Each row in the table either represents a different model and/or the ensemble approach with different strategies for the weights definition. The first row corresponds to the Ground Truth (GT) model, a standard model‐based classifier trained using ground truth labels. This model serves as a benchmark for assessing the performance of all the other approaches relative to the “oracle.” The subsequent seven rows correspond to the base learners. That is, standard model‐based classifiers separately trained using the labels provided by the M=7 annotators. Continuing toward the bottom of the table, we gather comprehensive information about ensemble models under different strategies for weights generation. Each model is associated with three distinct accuracy values depending on the considered covariance structure, denoted by LDA, QDA, and EDDA, respectively. As expected, the model fitted with ground truth labels has the best overall performance, while the base learners linked to experts achieve higher levels of accuracy than those linked to novices. Among the base learners, the best model has an accuracy of 0.745 and an SD of 0.12. This result is obtained with the labels of the fourth annotator and with an LDA covariance structure. Given that in Scenario 1 we set $\Sigma g=I_{3}\;\forall g=1,\text{…},3$, it goes without saying that LDA would emerge as the optimal selection for a base learner. As we direct our focus toward evaluating the ensemble models, a noteworthy observation emerges: the consistent superiority of the ensemble models compared to the individual base learners, irrespective of the chosen strategy for the determination of the weights. Particularly, the EN ensemble model stands out as the most effective among the configurations. However, it is essential to acknowledge that in real‐world scenarios the level of expertise is often not readily discernible. Consequently, there are situations where EN might not be applicable. In such cases, the data‐driven ensemble models surface as highly favorable alternatives. Demonstrating their prowess, the MV and the first variant of the Iterative Algorithm (ItAlg1) approaches achieve an impressive accuracy rate above 0.75. Remarkably, this accuracy surpasses that of the most proficient expert base learner. This accomplishment is observed when utilizing either LDA or EDDA covariance models for base learners. A salient advantage of the data‐driven models is their agnostic nature toward prior knowledge, making them adaptable across diverse scenarios. The compelling alignment between the performance of the data‐driven approaches and EN casts light on the efficiency of the scoring algorithms, adopted in MV, ItAlg1, and ItAlg2, in distinguishing between the two level of expertise of the considered annotators. For an in‐depth exploration of this aspect, refer to Section 4.2.2. Regarding the covariance structure, across the ensemble models accuracies exhibit a general tendency to be higher when an all‐equal LDA approach is employed. This choice entails the imposition of equal covariance matrices on the base learners, which is the structure used to generate the synthetic data. It is legitimate to question whether the efficacy of staking simple models closely ties to this specific context. All‐different QDA covariance structures might excel in scenarios where data exhibit more intricate patterns. However, the combination of such models could potentially lead to overfitting. In contrast, EDDA is anticipated to offer heightened flexibility as it is able to adjust the selection of the covariance model by evaluating all possible candidates. A deeper understanding of the problem is achieved by looking at the results related to Scenario 2 (see Figure 2b), in which the features possess class‐wise different correlation patterns. Accuracy results are reported in the second section of Table 4. The higher accuracy of the GT model fitted with a QDA covariance structure is indicative of the correct identification of the modeling choice in Scenario 2. When transitioning from QDA to LDA and EDDA, there is a decrease, from 0.88 to 0.859 and 0.871, respectively. An opposite trend emerges for the base learners, whose labels are affected by noise. QDA covariance model ceases to be the most suitable choice. The base learners utilizing a QDA covariance structure exhibits overfitting tendencies. This is characterized by the model excessive adaptation to the noise present in the labels, causing a decline in its ability to generalize to unseen data points, ultimately compromising its predictive performance. The problem is exacerbated as they are combined into an ensemble model. Indeed, when analyzing the bottom of the table, it becomes evident that LDA and EDDA covariance structures attain higher accuracy values. Reflecting on the insights from both scenarios, a discernible trend emerges: combining simpler models results in an ensemble configuration with improved robustness, less susceptible to overfitting. It is important to note that while this offers valuable guidance, it is not a definite rule. Different data sets with varying levels of noise might exhibit distinct benefits from combining more complex models. Lastly, note that similar to what is observed in the first scenario, for Scenario 2, irrespective of the increased data complexity, all ensemble models consistently outperform the individual base learners. The same outcome is observed when examining the predictive performance of the three competing methods presented in Table 5. Specifically, all methodologies achieve an average accuracy that remains around 0.7, with Dawid and Skene (1979), Raykar et al. (2010), and Rodrigues et al. (2013) performing even worse than the single‐best expert annotator. A possible explanation for this behavior is that all methods rely on multiclass Logistic Regression as the base classifier, which differs significantly from the generative model used for simulating the synthetic data. A more coherent result with the proposed methodology, albeit still lower in terms of accuracy, is found when examining the real‐data scenario (see Section 5).

**FIGURE 4:**
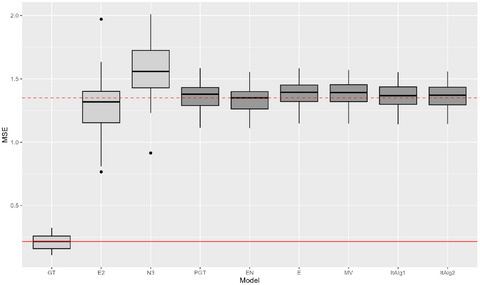
Boxplot of the mean squared errors for the S=50 simulations in Scenario 1. The light gray boxes denote, in order, ground truth model and two base learners with expert and novice annotators. Dark gray identifies the ensemble models with different weights specification. Red horizontal line highlights the median value of ground truth MSE, while dashed red line is located at the height of median MSE of EN ensemble model.

**FIGURE 5:**
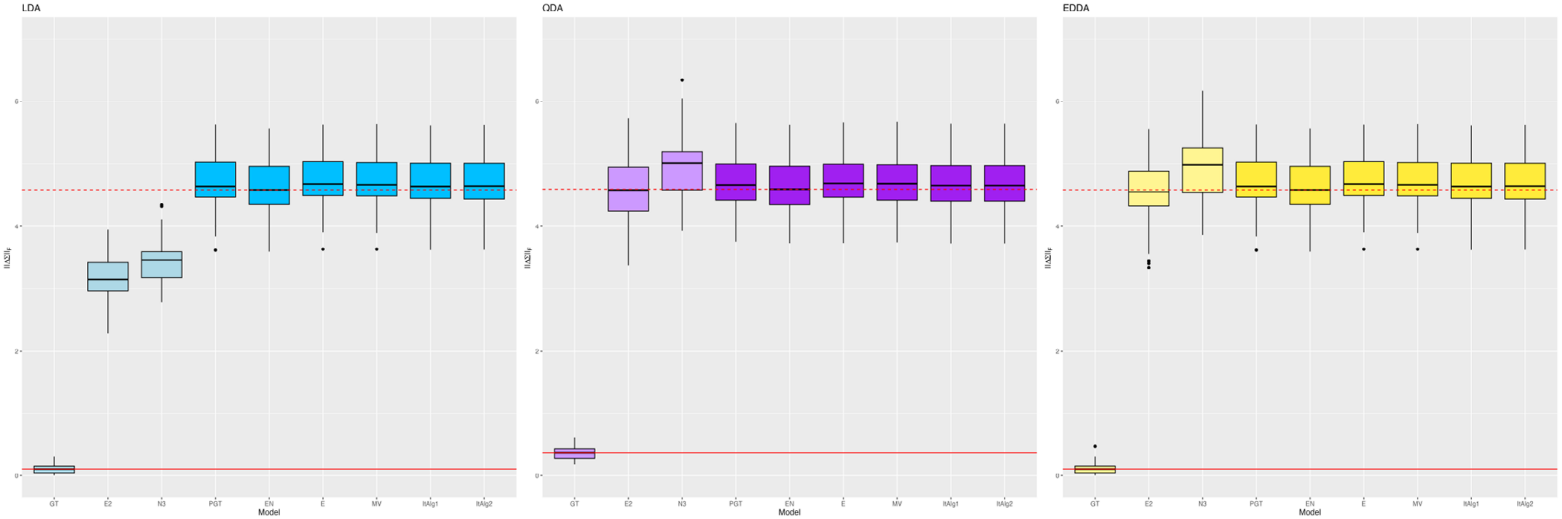
Boxplot of the covariance error ${\left||\Delta \Sigma |\right|}_{F}$ for the S=50 simulations in Scenario 1. Two shades of blue identify all‐equal covariances (LDA), shades of purple identify all‐different covariances across classes for the base learners (QDA) while shades of yellow identify BIC covariance structure selection (EDDA). The light hue is used for ground truth model and base learners, the dark hue for ensemble models with different weights specification. Red horizontal line highlights the median value of ground truth covariance error, while dashed red line is located at the height of median covariance error of EN ensemble model.

We now shift our attention toward the ability of the ensemble model in correctly retrieving the parameters used to generate the Gaussian classes. To do so, we introduce the second set of evaluation metrics which are focused on quantifying the goodness of parameters estimation. Specifically, we look at how well the different models retrieve means and covariance matrices of the G=3 classes. We utilize the Euclidean norm to measure the distance between the true mean vectors $\mu_{g}$ and the estimated ones ${\hat{\mu }}_{g}$ for each group g=1,2,3. We then average these distances defining the mean squared error (MSE) metric. Similarly, to evaluate errors in the covariance matrices estimation, we employ the Frobenius norm. By computing the difference between the true covariance matrices $\Sigma_{g}$ and the estimated ones ${\hat{\Sigma }}_{g}$ for each group g=1,2,3, we obtain a matrix $\Delta \Sigma_{g}$ upon which the Frobenius norm is applied. By averaging these norms, we construct an aggregated error metric that assesses the covariances estimation. We compute averages across the S=50 simulation runs for both metrics as follows:

(15) $$\begin{array}{cc} \text{MSE} & =\text{avg}\left(\frac{1}{G}\sum_{g=1}^{G}||\Delta \mu_{g}|{|}_{2}\right), \end{array}$$

(16) $$\begin{array}{cc} \left||\Delta \Sigma \right|{|}_{F} & =\text{avg}\left(\frac{1}{G}\sum_{g=1}^{G}||\Delta \Sigma_{g}|{|}_{F}\right), \end{array}$$

where $\Delta \mu_{g}=\mu_{g}-{\hat{\mu }}_{g}$ and $\Delta \Sigma_{g}=\Sigma_{g}-{\hat{\Sigma }}_{g}$ for each g=1,…,G where the estimated quantities vary with respect to the chosen method and/or weights definition strategy. Figures 4 and 5 report boxplots of the empirical MSE and covariance error distribution across the S=50 runs for Scenario 1. For average MSE and covariance error values for both Scenarios 1 and 2 refer to Table 4. A total of 14 competitors are analyzed but, to make the plot more interpretable, we have selected to depict only 9. More specifically, we report a base learner representative of the expert annotators' category and another representative of novices. It is immediately noticed that, as expected, the ground truth errors are close to zero for both means and covariance matrices. A more intricate comparison emerges when analyzing the base learners and the ensemble models. The boxes of the latter are located between the base learners, the expert box has a slightly lower median value, and the novice box has a higher median value. We also notice that ensemble models achieve parameter estimation performances that fall between those of experts and novices, but showcasing reduced variability. The three plots in Figure 5 illustrate the covariance error using blue, purple, and yellow colors for the LDA, QDA, and EDDA covariance structure, respectively. Across all cases, the median values for the GT are consistently positioned close to zero. Overall, we observe slightly lower values for LDA and EDDA compared to QDA structures. Base learners with an LDA covariance are notably distinctive for exhibiting lower error, both expert and novice base learners estimate covariance matrices more accurately than in the cases where QDA and EDDA are employed. In this scenario, the LDA model demonstrates strong performance due to its alignment with the true covariance structure in the data‐generating process. Nevertheless, when comparing the ensemble models with LDA, QDA, and EDDA, identified by the darker shade, we do not find consistent confirmation of this pattern. This phenomenon arises because, even when using LDA base learners, the resulting ensemble covariances are not necessarily equal across the G classes, as the all‐equality property is lost during the weighted average phase. In summary, the error for ensemble models using LDA is greater than that for base learners but comparable to the error of other ensemble models. Once again EN stands out, being the ensemble model with lowest median error. The same analysis is repeated for Scenario 2, in which the covariance matrices are different across classes. The numerical results are reported in Table 4. The MSE values exhibit the same trend as in Scenario 1, with ensemble model placed between experts and novices. Results for covariance errors present a different picture: the GT model estimate very well the covariance matrices if QDA and EDDA covariance models are selected, otherwise the error is high. This may be expected, with the GT model always showcasing superior performance when the correct covariance structure is set during model training in a noise‐free context. However, when noise is added to the labels the error escalates even more significantly for QDA and EDDA: using LDA base learners results in an error increase, while combining QDA or EDDA base learners leads to ensemble models with errors that are closely aligned to base learners errors.

#### 4.2.2 Scoring System Results

**FIGURE 6:**
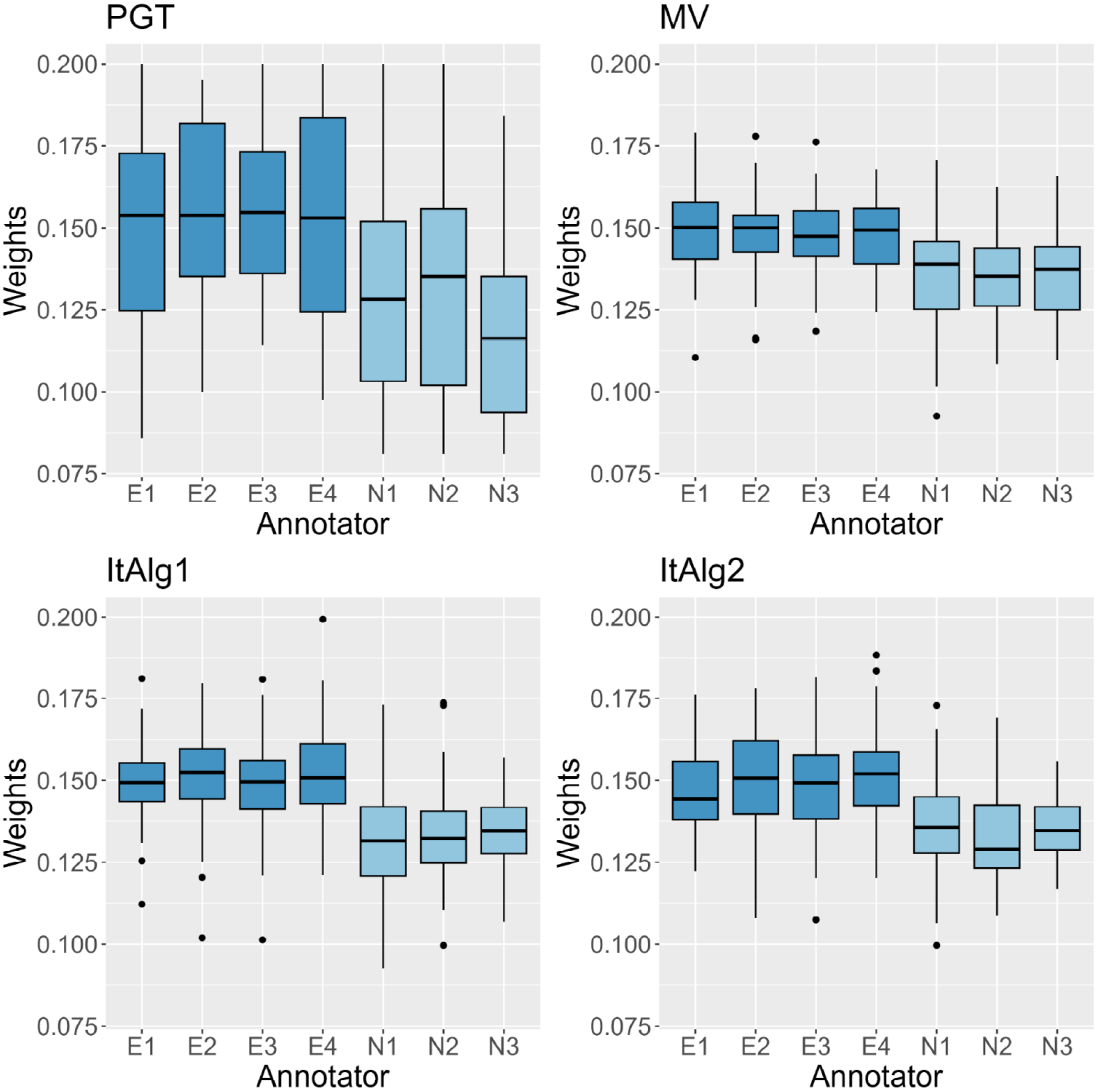
Annotators scores boxplot in Scenario 1. The darker shade of blue identifies expert annotators, while the lighter shade of blue identifies novice annotators. The subplots, in order, correspond to PGT, MV, and both versions of ItAlg.

Among the discussed strategies for weights generation, some offer a broader utility beyond generating classification outcomes by providing scores designed to evaluate annotator accuracy. Specifically, we are referring to PGT, MV, ItAlg1, and ItAlg2. The PGT model employs a scoring system that leverages a subset of observations for which ground truth is available. The data‐driven scoring systems of MV and ItAlg make assessments without necessitating any type of prior knowledge. These approaches possess the capability to extract sufficient information from the data to effectively discern the expertise associated to each annotator. In our simulated setting, we consider two types of annotators: four experienced and three more prone to labeling errors. In detail, annotators belonging to the former group are denoted by indexes m=1,2,3,4, while the latter are indicated by indexes m=5,6,7. To gauge the effectiveness of the scoring systems in distinguishing between experts and novices, in Figure 6 we display boxplots representing the empirical distribution of the scores obtained in the S=50 simulation runs for Scenario 1. In the four plots, the expert annotators showcase higher weights, demonstrating the scoring systems capability to accurately distinguish between experts and novices. However, a noticeable disparity is evident in the variability associated to the empirical distributions of the weights. Particularly, the PGT strategy exhibits greater variability that can be attributed to the fact that, in each simulation run, a subset of the data (approximately 10%) is randomly sampled, wherein the true labels are known. As a result, the PGT model scores encounter more variation due to this subset influence on the overall scoring distribution. In contrast, MV, ItAlg1, and ItAlg2 have very similar distributions, with a clear distinction between expert and novice annotators. A final note concerns ItAlg2. While it is inherently similar to ItAlg1, the figure shows a less clear distinction and generally more dispersion.

The simulation results have revealed both the strengths and weaknesses of the ensemble approach. When we examine accuracy values, it becomes evident that ensemble models exhibit robust predictive capabilities, outperforming the accuracy achieved by individual base learners as well as state‐of‐the‐art methods available in the literature. However, a limitation emerges when it comes to capturing the underlying data distribution. Despite their predictive power, the ensemble learner struggles to accurately recover the true underlying parameters generating the data. This duality, where strong predictive performance coexists with estimation bias can be explained by the concept of diversity in ensemble learning. It is widely accepted that ensembles perform best when their individual members provide a diversity of predictions. Quoting Goodfellow et al. (2016): “The reason that model averaging works is that different models will usually not make all the same errors on the test set.” More recently, Wood et al. (2023) present a unified theory on ensemble diversity for popular ensemble methods such as Bagging, Boosting, and Random Forests. Diversity can be incorporated through various means, and in the context of multiple sets of noisy labels herein discussed, it naturally arises from the utilization of distinct sets of noisy labels for each base learner. From our analysis, it emerges that diversity enhances the predictive performance, but on the other hand it does not improve parameters estimation.

All in all, thanks to the data‐driven weight generation procedures we are not only able to improve the classification accuracy but, as a by‐product of the learning process, we can also disentangle the degree of expertise of the annotators involved in the study. This is not only achieved in synthetic settings but also in a real‐case study, as reported in the next section.

## 5 Application on Gastroenterology Data

The considered real‐data application concerns the identification of gastrointestinal lesions by means of regular colonoscopic videos (Mesejo et al. 2016). In detail, a group of M=7 clinicians (comprising four experts and three novices) were tasked to review the recording for N=76 patients, providing assessments to determine whether the lesions were benign or malignant. The data set is publicly available in the University of California Irvine Machine Learning data repository (https://archive.ics.uci.edu/dataset/408/gastrointestinal+lesions+in+regular+colonoscopy).

A regular colonoscopy, a medical procedure used to examine the inside of the colon (large intestine) and rectum, is performed to detect any abnormalities or lesions. In this study, we are interested in identifying particular lesions called polyps. Polyps are small, noncancerous growths that can potentially develop into cancer over time. A regular colonoscopy may not be sufficient for the diagnosis of some small polyps or early signs of malignancy. Gastroenterologists may thus decide to use more sophisticated procedure such as chromoendoscopy, a specialized technique used during endoscopic to enhance the visualization of the mucosal lining of the digestive tract. Such a procedure involves the application of dyes or stains to the surface of the mucosa to highlight subtle changes, lesions, or abnormalities that might be difficult to see with standard white‐light endoscopy alone. Indigo carmine is one of the dyes commonly used in chromoendoscopy procedures. It is a blue dye that can be applied to the mucosa, and it helps to provide contrast and improve the differentiation between different types of tissue. Chromoendoscopy with indigo carmine can be especially useful for detecting and characterizing flat or subtle lesions, which might be missed by standard white‐light endoscopy. It is often used in surveillance of patients with conditions that increase the risk of gastrointestinal cancers, such as certain genetic syndromes. While chromoendoscopy can enhance diagnostic accuracy, it is also time‐consuming due to the need for dye application, observation, and potential biopsy. The objective of the analysis is to evaluate the efficacy of the ensemble model in diagnosing gastrointestinal lesions from standard colonoscopic videos, thus eliminating the necessity to resort to more invasive procedures.

Following the same preprocessing outlined in Ahfock and McLachlan (2021), out of the 698 original video features we focus on p=5 standardized variables (V113, V173, V475, V489, and V603) obtained by employing sparse discriminant analysis (Clemmensen et al. 2011). Specifically, the first two variables pertain to 2D textural characteristics: V113 corresponds to an autocorrelation homogeneous texture (AHT) feature, which offers insights into the lesion texture and pattern, while V173 is associated with Rotational Invariant Local Binary Pattern, a texture descriptor that remains unaffected by rotation and captures local patterns. The subsequent variables, V475 and V489, encompass Color Gray‐level Co‐occurrence attributes. These variables are instrumental in comprehending the color patterns and relationships intrinsic to the lesion. Lastly, the fifth variable, V603, represents a 3D shape feature, specifically derived from kernel principal component analysis (KPCA, Schölkopf et al. 1997). KPCA is a technique often employed to capture intricate patterns and complex relationships within data, making it a valuable tool for analyzing the 3D shape of the lesions. All in all, the selected variables encapsulate crucial characteristics of the lesions, encompassing texture, color attributes, and its 3D shape.

**FIGURE A.1:**
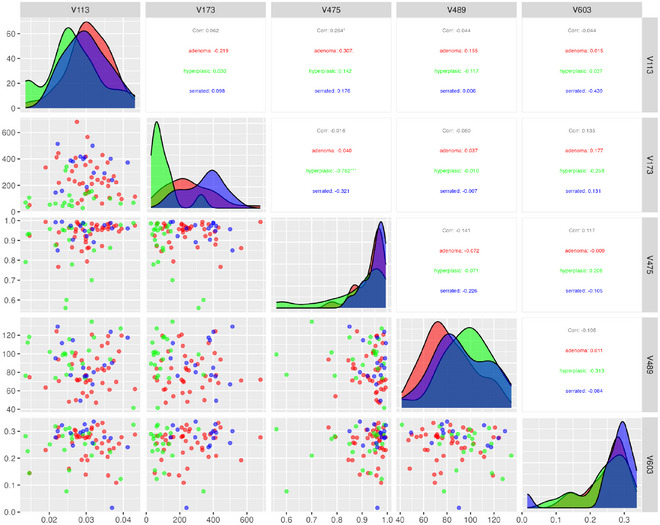
Pair plot of the features considered in the gastrointestinal lesions data set analyzed in Section 5. Red, green, and blue identify serrated, hyperplastic, and adenoma classes, respectively. On the main diagonal, class‐wise univariate densities are displayed. Bivariate scatterplots are reported in the lower panels while the upper panels report the sample correlation among features in the three classes.

The instances are manually classified into G=3 distinct polyp types: hyperplastic, adenoma, and serrated adenoma. Adenomatous polyps are the types most likely to develop into cancer if left untreated. They are commonly found in the colon and rectum. Hyperplastic polyps are typically benign, but there is a specific subtype known as serrated, the third class in the data, that have a slightly increased potential for progression to cancer, especially when they are large in size or found in certain locations in the colon. Hyperplastic lesions belong to the class “benign” while the adenoma and serrated adenoma can be regarded as “malignant” polyps. Alongside the noisy labels provided by the seven annotators ground truth labels are also available with a total of 15 instances of serrated adenomas, 21 of hyperplastic lesions, and 40 of adenomas. Refer to Appendix A, figure A1 for a visual representation of the data set. The primary aim of the study is to maximize accuracy, ensuring the most precise classification of these lesions leveraging on the set of noisy labels: results are reported in the next section.

### 5.1 Classification Performance

The predictive performance is assessed through a training‐test split of the N=76 units. The models are trained on a set with a sample size of 50, and the accuracy is then calculated on the remaining data, forming the test set. We repeat the analysis for 50 different train‐test splits. The average accuracy values for the ground truth model, single base learners, the proposed ensemble models, and the competing methods introduced in Section 4.1.3 are reported in Table 6. In addition, as an anonymous reviewer pertinently suggested, we have also included the localized kernel alignment‐based annotator relevance analysis (LKAAR), recently proposed in Gil‐Gonzalez et al. (2021), in our comparison to address potential clinicians interdependencies. Specifically, LKAAR is a kernel‐based method that models both the dependencies among annotators and the relationship between input features and labelers' performance through a nonparametric approach, demonstrating particularly strong performance when handling inconsistent labelers (Gil‐Gonzalez et al. 2021).

**TABLE 6:** Average accuracy on the test set for 50 training‐test splits of the Gastroenterology data, varying covariance structure, weights generation procedure, and ensemble strategy for the model proposed in Section 2 and for the competing models described in Dawid and Skene (1979), Raykar et al. (2010), and Rodrigues et al. (2013). Standard deviations in parentheses.

**Accuracy ACC (SD)**								
Ground truth model and base learners								
	**GT**	**E1**	**E2**	**E3**	**E4**	**N1**	**N2**	**N3**
**LDA**	0.595 (0.08)	0.579 (0.08)	0.574 (0.08)	0.542 (0.09)	0.581 (0.07)	0.548 (0.08)	0.614 (0.08)	0.531 (0.09)
**QDA**	0.535 (0.08)	0.519 (0.08)	0.576 (0.09)	0.496 (0.10)	0.505 (0.08)	0.451 (0.09)	0.569 (0.07)	0.456 (0.11)
**EDDA**	0.595 (0.08)	0.579 (0.08)	0.574 (0.08)	0.542 (0.09)	0.581 (0.07)	0.548 (0.08)	0.614 (0.08)	0.531 (0.09)
Ensemble models								
		**PGT**	**EN**	**E**	**MV**	**ItAlg1**	**ItAlg2**	
**LDA**		0.652 (0.06)	0.647 (0.06)	0.645 (0.07)	0.645 (0.06)	0.652 (0.06)	0.651 (0.06)	
**QDA**		0.531 (0.10)	0.507 (0.11)	0.532 (0.10)	0.530 (0.10)	0.528 (0.10)	0.528 (0.10)	
**EDDA**		0.652 (0.06)	0.647 (0.06)	0.645 (0.07)	0.645 (0.06)	0.649 (0.06)	0.648 (0.06)	
Competing models								
	**Dawid and Skene**		**Raykar**		**Rodrigues**		**LKAAR**	
	0.598 (0.11)		0.565 (0.11)		0.642 (0.07)		0.574 (0.09)	

Upon initial examination of Table 6, it is evident that utilizing either LDA or EDDA covariance structure results in higher accuracy with respect to QDA. The first three rows clearly indicates that QDA is susceptible to overfitting, a drawback that persists after the creation of the ensemble models. Having conducted this preliminary screening, our subsequent investigations will concentrate solely on the LDA and EDDA cases. Contrary to what one might think by noting that the accuracy values of the base learners are identical for LDA and EDDA covariance model, the covariance structure selected through the BIC employed by EDDA does not always align with the all‐equal one imposed by LDA. Throughout the 50 runs, we recorded the chosen covariance model by EDDA. Most frequently, we encountered the EEI model, followed by less frequent occurrences of VVI and EVI. Notably, all three belong to the family of diagonal covariance structures. The preference of EDDA for models with covariance structures featuring fewer parameters, such as EEI, does not compromise the accuracy of either base learners or ensemble models. Observing the fourth to sixth rows of Table 6, a clear pattern emerges: the ensemble models achieve higher accuracy values compared to the base learners and, remarkably, the ground truth model, irrespective of the strategy employed for determining the weights. Particularly noteworthy are the PGT and the ItAlg models, which boasts the highest accuracy value of 0.65. Nonetheless, recall that the applicability of PGT is contingent upon the availability of ground truth labels. Surprisingly, the MV and EN approaches appear to face greater challenges in accurately predict test samples. In fact, they exhibit a lower accuracy rate of 0.64, which is the same as that achieved by the equal weights model E. The limitations of the latter model performance were already evident in the simulation context. The assumption of equal weights, due to the difference in expertise of the considered annotators, adversely impacts predictive performance. This trend only partially applies to the competing methods included in the study. Notably, the methodology by Rodrigues et al. (2013), which explicitly models the annotators' expertise with latent variables, achieves performance only slightly lower than our proposal. Contrarily, the flexibility entailed by the nonparametric LKAAR model does not result in an improvement in predictive accuracy, with performance comparable to simpler methods such as those by Dawid and Skene (1979) and Raykar et al. (2010). All things considered, along with a slight improvement in prediction accuracy, the flexibility provided by the options for determining annotators' weights makes our proposal the most favorable for classifying gastrointestinal lesions from annotators with varying degrees of expertise. Related to that, a noteworthy insight on this matter arises from the base learners comparison. The highest accuracy value, that is 0.614, is achieved by the model linked to the sixth annotator, who falls within the novice class. This revelation implies that information about annotators expertise levels might not be as reliable as presumed.

**FIGURE 7:**
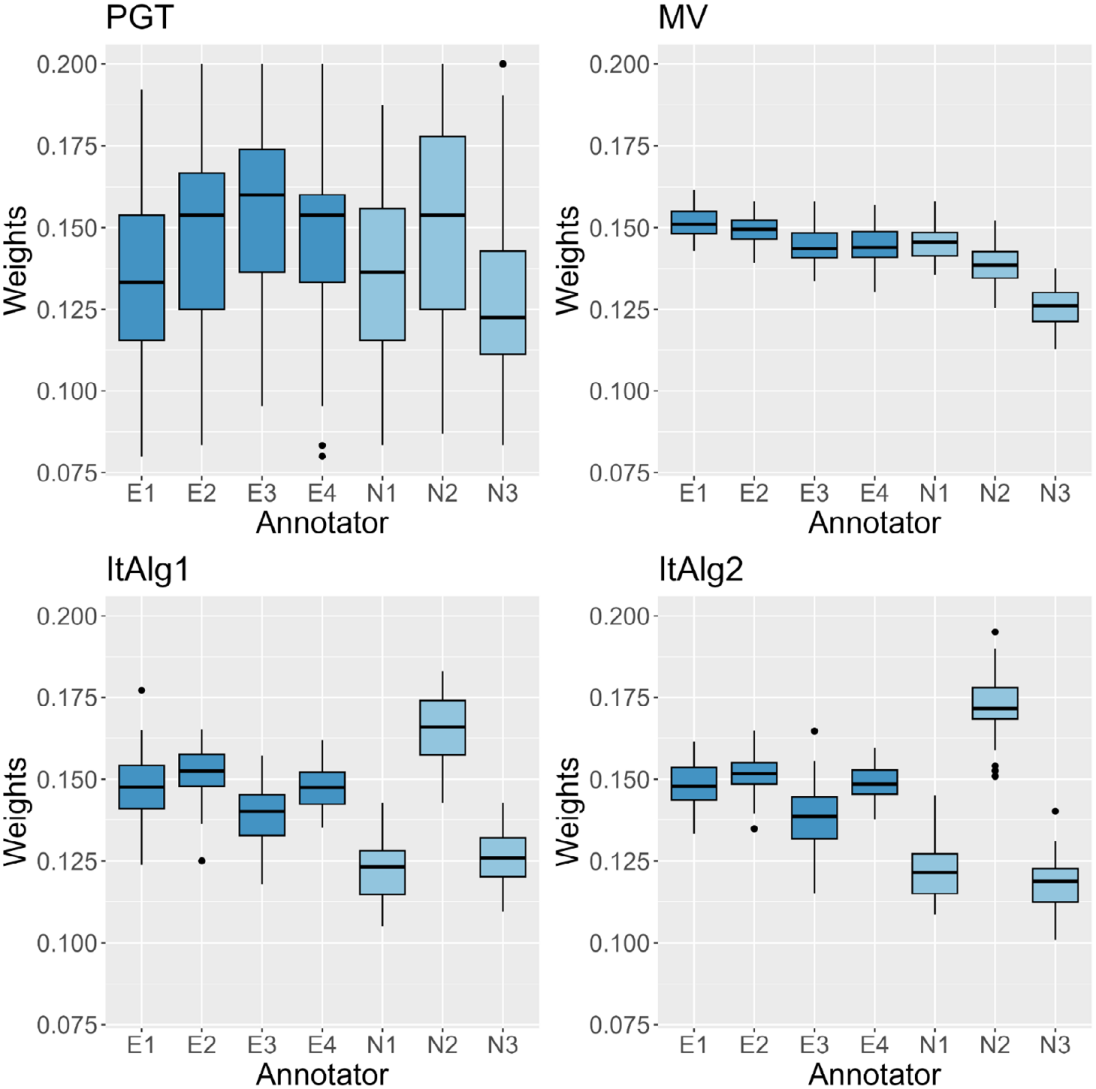
Boxplots of the empirical distribution of annotators' weights for 50 repetitions of the training‐test split, employing various weight determination strategies. The darker shade of blue identifies expert annotators, while the lighter shade of blue identifies novice annotators. The subplots, in order, correspond to PGT, MV, and both versions of ItAlg weights determination strategies.

**FIGURE 8:**
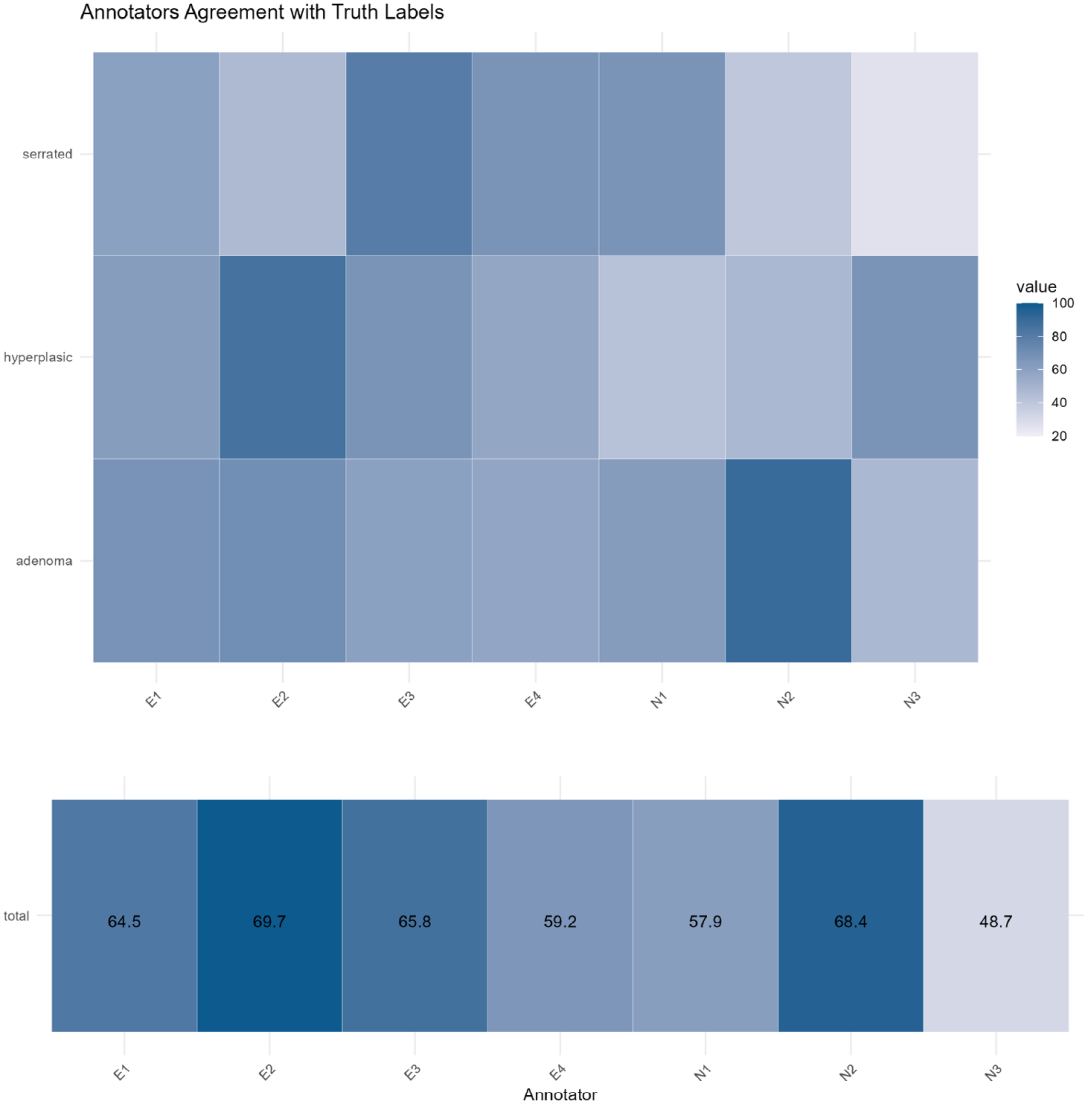
Categorical heatmap visualizing agreement between annotators and ground truth labels. The heatmap employs a graded color scale ranging from white to blue to depict the degree of agreement between the noisy labels and the ground truth. Each cell showcases the percentage of agreement, with darker shades indicating higher alignment and lighter blues highlighting areas of disparity.

To further explore the uncertainties that have emerged regarding the annotators set, we undertake a comprehensive analysis of the outputs generated by the scoring systems. The boxplots illustrating this analysis are presented in Figure 7. Concerns about the reliability of annotators' expertise a priori information find partial validation through the boxplots. Specifically, the PGT and both ItAlg models indicate the sixth box as having the highest median value. The PGT boxes display significant dispersion, aligning with findings from the simulation context. When comparing the boxes generated by ItAlg, a clear distinction emerges between the experts and the first and third novices. However, the second novice appears to be inaccurately identified as inexperienced. In contrast, the scoring system based on MV presents a different pattern. This system assigns comparable weights to all seven annotators, with only a slight reduction for the novice annotators. Given the significant difference highlighted by ItAlg and considering the relatively uniform scores of MV, accompanied by a relatively low accuracy value, it was deemed necessary to reconsider the assumptions regarding the expertise level of annotators. In particular, to get a feedback on the accuracy of annotations, we compared annotators' labels with ground truth. The heatmap in Figure 8 highlights, with cells of a darker shade, annotators, and classes for which label accuracy is high. Crossing the position of the sixth annotator and the “adenoma” class, we observe high accuracy in N2 annotations for the most numerous class. Given the class imbalance, achieving high accuracy on the majority class “adenoma” has a significant impact on the overall accuracy. Indeed, from the bottom row we can observe that N2 has an accuracy of 68%, close to that of the best expert. Thus, a decision was made to reevaluate the EN model considering also the sixth annotator as expert. This reassessment results in an increase in accuracy from 0.645 to 0.68, which currently represent the best performance achieved in the analysis.

### 5.2 Best Ensemble Performance Evaluation for Clinical Practice

In the pursuit of optimizing predictive accuracy, we have identified EN as the most promising ensemble classifier. In what follows, we motivate the employment of the ensemble model in clinical practice. First, it is worth noting that while adenoma and serrated adenoma represent distinct types of polyps, they both necessitate removal as part of the medical protocol. The classification of a serrated polyp as an adenoma thus does not alter the procedural requirements. Nonetheless, wrongly classifying either of them as hyperplastic would entail the misdiagnosis of malignant lesion. To elaborate on the type of error being made, we can consolidate adenoma and serrated adenoma into a single entity termed “malignant” as they denote polyps requiring removal. By doing so, we reduce the categories to a binary classification setup, enabling the utilization of traditional metrics employed in binary scenarios. We first analyze the false negative rate, that is, number of adenoma and serrated adenoma that are classified as hyperplastic. In particular, we resort to sensitivity and negative predicted value (NPV). Sensitivity, or Recall, quantifies the ratio of accurate positive predictions (adenoma or serrated adenoma correctly identified as malignant) to all actual positive cases. A high sensitivity signifies that the model excels at identifying true positive cases, which encompass adenoma and serrated adenoma. On the other hand, NPV calculates the proportion of true negative cases (hyperplastic correctly identified as such) out of all instances predicted as negative. In practical terms, a high NPV implies that when the model predicts an individual as negative, the probability of them genuinely not having the condition is substantially high. In addition to these primary metrics, an auxiliary set of evaluation measures is introduced to gauge the occurrence of true negatives and false positive. In this context, the impact of false positive is of relatively lower concern for medical professionals. The surgical removal of a hyperplastic polyp is less critical. The chosen focal metrics for assessment is specificity and precision. A high specificity value indicates that the model is proficient in accurately identifying true negative instances. Meanwhile, a high precision value assures that the model positive predictions are reliable and accurate. In Table 7, these additional four metrics are reported when a binary classification setting is considered. Given the high values of both sensitivity and precision it is now clear that the false negative rate is low. Specificity and NPV suggest that hyperplastic is more easily misclassified, resulting in resection of nonmalignant polyps.

**TABLE 7:** Sensitivity, NPV, specificity, and precision obtained in the binary setup for the ensemble model EN. Standard deviations in parentheses.

	**Sensitivity**	**NPV**	**Specificity**	**Precision**
**EN**	0.89 (0.06)	0.74 (0.12)	0.76 (0.14)	0.91 (0.05)

## 6 Conclusion and Future Research Direction

Performing supervised classification of medical data with noisy labels is a formidable task. This challenge arises frequently due to a multitude of factors that encompass the intricacies of medical practice, the variability in human annotation, and the inherent uncertainty in healthcare analytics. In this manuscript, we have introduced an ensemble model‐based classifier designed to effectively manage multiple sets of noisy labels, specifically suited for situations in which both annotators and data samples may be scarce. Our contribution has encompassed the definition of the stacking procedure and the provision of six distinct strategies for determining the annotators weights. These strategies have been broadly classified into two categories, with two methods requiring specific information for their implementation, while the remaining options fall within a data‐driven framework, providing users with a spectrum of choices. An initial approach relies on a subset of known ground truth labels, while the second strategy necessitates knowledge of the annotators' level of expertise. When no a priori information is available, the use of entirely data‐driven alternatives is essential. The choice of equal weights has provided a simple and direct approach. Alternatively, strategies involving distinct scoring systems have also been proposed. By yielding weights that directly align with the competence of each annotator, the unknown level of expertise has been inferred as a by‐product of the learning process. Through both simulations and a real‐data application, notable improvements in predictive performance have been observed compared to using single sets of noisy labels and state‐of‐the‐art alternatives.

The devised methodology also possesses limitations. Primarily, it is assumed that annotator‐wise each class can be conveniently modeled through a Gaussian distribution. While we acknowledge that our method could be effectively employed in contexts where the data distribution is not strictly Gaussian, enabling the construction of an effective ensemble decision rule using Gaussian densities, the achieved flexibility may not be adequate when the relationships between features and classes are complex or highly nonlinear. A potential option would be to utilize mixture‐based discriminant analysis (MclustDA, Fraley and Raftery 2002) as base learners, enabling the approximation of arbitrarily complex decision boundaries. While this approach may seem promising, integrating MclustDA models separately fitted to each annotator presents a significant difficulty and nontrivial merging solutions, such as those proposed in Glodek et al. (2013) and Casa et al. (2021), must be adopted to address this issue effectively.

Building upon the findings and limitations of the current work, several promising directions for future research naturally arise. First, the proposed method could be extended to also handle attribute noise, along the lines of Cappozzo et al. (2020b). Second, more flexible model‐based classifiers could be considered for base learners, such as t‐distributions (Andrews et al. 2011; Andrews and McNicholas 2012), generalized hyperbolic mixtures (Morris and McNicholas 2016), and skewed power exponential distributions (Dang et al. 2023). Lastly, it may be of interest to deal with high dimensional features, for which mixtures of factor analyzers and extensions (McLachlan et al. 2003; McNicholas and Murphy 2008; Murray et al. 2014; Lin et al. 2016) as well as parsimony‐inducing covariance structures (Bouveyron et al. 2007; Bouveyron and Brunet 2012; Cavicchia et al. 2022, 2024) can prove fruitful alternatives. Some options are currently being explored and they will be the object of future research.

## Disclosure

The authors have nothing to report.

## Conflicts of Interest

The authors declare no conflicts of interest.

### Open Research Badges

This article has earned an Open Data badge for making publicly available the digitally‐shareable data necessary to reproduce the reported results. The data is available in the Supporting Information section.

This article has earned an open data badge “**Reproducible Research**” for making publicly available the code necessary to reproduce the reported results. The results reported in this article could fully be reproduced.

## Supporting information

Supporting Information

## Data Availability

The data that support the findings of this study are available in UCI Machine learning repository at https://archive.ics.uci.edu/dataset/408/gastrointestinal+lesions+in+regular+colonoscopy. These data were derived from the following resources available in the public domain: ‐ Gastrointestinal Lesions in Regular Colonoscopy, https://archive.ics.uci.edu/static/public/408/gastrointestinal+lesions+in+regular+colonoscopy.zip.
